# The Herpes Simplex Virus 1-Encoded Envelope Glycoprotein B Activates NF-κB through the Toll-Like Receptor 2 and MyD88/TRAF6-Dependent Signaling Pathway

**DOI:** 10.1371/journal.pone.0054586

**Published:** 2013-01-28

**Authors:** Mingsheng Cai, Meili Li, Kezhen Wang, Shuai Wang, Qiong Lu, Jinghua Yan, Karen L. Mossman, Rongtuan Lin, Chunfu Zheng

**Affiliations:** 1 Institute of Biology and Medical Sciences, Soochow University, Suzhou, China; 2 Wuhan Institute of Virology, Chinese Academy of Sciences, Wuhan, China; 3 Department of Pathogenic Biology and Immunology, Guangzhou Medical University, Guangzhou, China; 4 Department of Veterinary Medicine, Foshan Science and Technology University, Foshan, China; 5 CAS Key Laboratory of Pathogenic Microbiology and Immunology, Institute of Microbiology, Chinese Academy of Sciences, Beijing, China; 6 The Department of Pathology and Molecular Medicine, Institute for Infectious Disease Research, McMaster University, Hamilton, Canada; 7 Lady Davis Institute for Medical Research, Jewish General Hospital and Department of Medicine, McGill University, Montreal, Canada; University of Pittsburgh School of Medicine, United States of America

## Abstract

The innate immune response plays a critical role in the host defense against invading pathogens, and TLR2, a member of the Toll-like receptor (TLR) family, has been implicated in the immune response and initiation of inflammatory cytokine secretion against several human viruses. Previous studies have demonstrated that infectious and ultraviolet-inactivated herpes simplex virus 1 (HSV-1) virions lead to the activation of nuclear factor kappa B (NF-κB) and secretion of proinflammatory cytokines via TLR2. However, except for the envelope glycoprotein gH and gL, whether there are other determinants of HSV-1 responsible for TLR2 mediated biological effects is not known yet. Here, we demonstrated that the HSV-1-encoded envelope glycoprotein gB displays as molecular target recognized by TLR2. gB coimmunoprecipitated with TLR2, TLR1 and TLR6 in transfected and infected human embryonic kidney (HEK) 293T cells. Treatment of TLR2-transfected HEK293T (HEK293T-TLR2) cells with purified gB results in the activation of NF-κB reporter, and this activation requires the recruitment of the adaptor molecules myeloid differentiation primary-response protein 88 (MyD88) and tumor necrosis factor receptor-associated factor 6 (TRAF6) but not CD14. Furthermore, activation of NF-κB was abrogated by anti-gB and anti-TLR2 blocking antibodies. In addition, the expression of interleukin-8 induced by gB was abrogated by the treatment of the human monocytic cell line THP-1 with anti-TLR2 blocking antibody or by the incubation of gB with anti-gB antibody. Taken together, these results indicate the importance and potency of HSV-1 gB as one of pathogen-associated molecular patterns (PAMPs) molecule recognized by TLR2 with immediate kinetics.

## Introduction

The innate immune response is an early line of host defense during infection. It is now known that viruses, similar to bacteria and fungi, are initially recognized by a class of host immune sensor molecules that are referred to as germline-encoded pattern recognition receptors (PRRs), via their encoded proteins containing evolutionarily conserved pathogen-associated molecular patterns (PAMPs). The Toll-like receptors (TLRs) are the most well-characterized family of PRRs, phylogenetically conserved from *Drosophila* to humans, and constitute a family of receptors that detect an increasingly broad range of pathogens that triggers a great deal of cellular responses [1].

To date, it has been shown that TLR2 plays a key role in the microbial antigen activation of nuclear factor kappa B (NF-κB) [2]. Signaling through TLR2/MyD88 (myeloid differentiation primary-response protein 88) activates NF-κB and promotes the production of proinﬂammatory cytokines such as interleukin 1 (IL-1), IL-6, IL-8, IL-12 and monocyte chemotactic peptide 1 [3]–[4]. In fact, TLR2 forms a heterodimer with its coreceptors TLR1 or TLR6, or perhaps other PRRs, for detection of various microbial components, and in some cases, neither TLR1 nor TLR6 is required for the molecular recognition [5]. TLR2 may also signal as a homodimer to recognize different types of ligands. Moreover, depending on the nature of the ligands, CD14, as a co-receptor of PRR for many different microbial antigens [6], is not absolutely required for all TLR2 signaling activity [7]. Thus far, a large body of work demonstrates that TLR2 recognizes structural components of several viruses [8]–[11], including members of the herpesvirus family, such as varicella-zoster virus (VZV), murine gammaherpesvirus-68, human cytomegalovirus (HCMV), and Epstein-Barr virus [12]–[15].

Several studies have implicated TLRs as important players during herpes simplex virus (HSV) infection, depending upon the cell types. Recent studies suggest that TLR2 signaling may be involved in innate responses to HSV [16], and the HSV-1-encoded envelope glycoprotein gH and gL are the specific viral proteins that can activate TLR2 signaling [17], however, whether there are other determinants of HSV-1 responsible for TLR2 mediated biological effects is not known yet. Interestingly, HCMV-encoded gB is reported to interact with TLR2 and this interaction is essential for initiating an inflammatory cytokine secretion [13]. This might also be the case for the possible involvement of HSV-1-encoded gB in the interaction with TLR2. In the present study, using TLR2-transfected human embryonic kidney (HEK) 293T (HEK293T-TLR2) cells and the human monocytic cell line THP-1, we provide pieces of evidence that HSV-1-encoded envelope glycoprotein gB can specifically activate cells via TLR2-dependent signaling, a process that may contribute to the production of inflammatory cytokines during HSV-1 infection.

## Materials and Methods

### Reagents and Virus

Yeast zymosan was purchased from Invivogen, polyinosinic-polycytidylic acid (poly(I:C)) was bought from Amersham Bioscience, heparinase and Lipopolysaccharide (LPS) from *Escherichia coli* serotype 011:B4 were obtained from Sigma-Aldrich, and LPS was re-purified by phenol extraction prior to use to remove contaminating lipopeptides, as described previously [18]. Cell culture media Dulbecco’s Modified Eagle’s Medium (DMEM) and RPMI1640 and fetal bovine serum (FBS) were purchased from Gibco. The wild-type (WT) HSV-1 (strain F) and HSV-1 BAC Luc (expressing firefly luciferase) [19] were multiplied, titered and purified as previously described [20]–[22].

### Cell Culture

HEK293T cells and Vero cells were maintained as adherent monolayers by serial passage in DMEM. Human monocytic cell line THP-1 (Cell Resource Center, Shanghai Institutes for Biological Sciences, Chinese Academy of Sciences) was grown in suspension in continuous culture with RPMI 1640 medium. All media mentioned above were supplemented with 10% (vol/vol) heat-inactivated FBS, L-glutamine (2 mM/L), penicillin (100 µg/ml), streptomycin (100 U/ml), essential sodium pyruvate (1%) and HEPES (10 mM/L) and cells were maintained at 37°C in a 95% air-5% CO2 humidified incubator.

### Plasmids

Human WT pCMV1-FLAG-hTLR2 expression vector and dominant-negative (DN) pCMV1-FLAG-DN-hTLR2, pcDNA3.1-DN-hTLR6 and pCMV1-FLAG-DN-hTLR1 expression vectors were kindly provided by Dr. Ruslan M. Medzhitov. pCMV1-FLAG-hTLR4, pEFBOS-HA-hMD2 and pcDNA3.1-hCD14 expression constructs were a generous gift from Dr. Stefanie N. Vogel. pHA-DN-MyD88, pFLAG-DN-TRAF2 and pDN-TRAF6 expression vectors were described previously [23]. pcDNA3.1-DN-hTLR2, pEFBOS-hTLR1-HA, pFLAG-TLR6, pTLR3-FLAG and pcDNA3-gB (HSV-1 encoded) expression plasmids were generous gifts from Drs. Shinichi Yokota, Shizuo Akira, Frederick J. Sheedy, Misako Matsumoto and Helena Browne, respectively. The NF-κB luciferase promoter construct pNF-κB-Luc and the transfection control reporter vector pRL-TK were purchased from Clontech Laboratories and Promega, respectively. The eukaryotic expression plasmids pcDNA3.1 (Invitrogen), pCMV-FLAG (Beyotime Institute of Biotechnology) and pCMV-HA (Beyotime Institute of Biotechnology) were purchased as indicated.

### Antibodies

Anti-human TLR2 monoclonal antibody (mAb) (IgG2a, clone TL2.1), IgG2a isotype control mAb, anti-IκBα rabbit polyclonal antibody (pAb), His-probe Alexa Fluor 488 (AF488) Ab, anti-gB (HSV-1 encoded) mAb and normal mouse IgG were purchased from Santa Cruz Biotechnology, mAbs against FLAG and hemagglutinin (HA) epitopes were purchased from Sigma. Anti-gB pAb R68 against the HSV-1-encoded gB was a gift from Drs. Gary H. Cohen and Roselyn J. Eisenberg.

### Protein Expression and Purification of the Soluble Protein of HSV-1-encoded gB

Soluble gB protein (gBs) of HSV-1 was prepared and purified as described previously [24]–[25]. Briefly, DNA corresponding to amino acid residues 30–730 of the ectodomain of the HSV-1 (strain F) gB was amplified and cloned into the baculovirus transfer vector pAcGP67-B (BD Biosciences) to allow for efficient secretion of recombinant protein, which containing an N-terminal his-tag, tetramerizing sequence and thrombin cleavage site. The recombinant baculovirus was prepared based on the manufacturer’s instructions (Invitrogen) and spodoptera frugiperda (Sf9) cells were infected with high-titer recombinant baculovirus at a multiplicity of infection (MOI) of 5. Then, the cell supernatant was applied to the HisTrap FF 5 ml column (GE Healthcare) and Superdex-200 10/300 GL column (GE Healthcare) for purification. Fractions containing highly-pure gBs were collected and concentrated using a membrane concentrator with a molecular weight cutoff of 10,000 (Millipore). The supernatants of Sf9 cell culture infected with recombinant baculovirus expressing influenza A virus hemagglutinin (HA) protein or baculovirus pAcGP67-B expressing no exogenous protein (mock baculovirus infection) were also prepared and purified similarly.

### Co-immunoprecipitation and Immunoblotting Analysis

Co-immunoprecipitation (Co-IP) experiments and immunoblotting (IB) analysis were performed as described previously [26]. All Co-IP experiments were repeated at least three times, and similar data were obtained.

### Soluble gB Binding to Anti-gB mAb

gBs reactivity to anti-gB mAb was measured by enzyme-linked immunosorbent assay (ELISA). gBs or baculovirus expressed HA (negative control) was immobilized on 96-well trays at a concentration of about 10 µg/ml in bicarbonate buffer for 16 h at 4°C or 2 h at 37°C. Unspecific binding sites were blocked with 5% bovine serum albumin (BSA) in phosphate-buffered saline (PBS) for 2 h at 37°C. Serial dilutions of anti-gB mAb (from 1∶3 to 1∶2187) were added to the wells in PBS containing 1% BSA and incubated for 1 h at 37°C. Unbound Abs were removed, and the bound Abs were reacted with anti-mouse Abs conjugated to peroxidase. The binding of gB and anti-gB was detected by incubation with tetramethyl benzidine (Thermo), and the optical density (O.D.) at 450 nm was read. The results are shown as the mean ± standard deviation (SD) of two independent experiments.

### Soluble gB Binding to Cells

To detect the cell binding capacity of gBs, HEK293T cells seeded in a 24-well plate were washed once with PBS and incubated with gBs (20 µg/ml) or anti-gB mAb (10 µg/ml) preincubated gBs for 0.5–1 h at 4°C. Then cells were washed three times with PBS and stained with His-probe AF488 Ab, a Alexa Fluor-conjugated mAb directed to the His tag engineered at the N terminus of truncated gB, in PBS containing 5% FBS for 1 h at 4°C. Control cells were incubated only with His-probe AF488 Ab. Cells were analyzed with a FACScalibur cytometer (Beckman Coulter). Heparinase treatment of cells was done as described previously [27].

### Inhibition of Viral Entry Experiment

To test the inhibition ability of viral entry by anti-gB mAb and to determin whether the soluble gB is a biologically functional form of the glycoprotein that able to inhibit virus entry, HEK293T cells seeded in a 12-well plate were incubated with purified gBs (20 µg/ml) for 2 h or left untreated. Then cells were mock infected or infected with HSV-1 BAC Luc or anti-gB mAb (10 µg/ml) preincubated HSV-1 BAC Luc (37°C for 1 h) at an MOI of 1 for 24 h. Following infection, cells were lysed with 200 µl of Passive Lysis Buffer (Promega), and firefly luciferase activity was assayed using the luciferase assay system (Promega) according to the manufacturer’s instructions. The results are shown as the mean ± SD of two independent experiments.

### Luciferase Reporter Gene Assays for NF-κB Activation

HEK293T cells were transiently cotransfected with selected expression plasmids by the standard calcium phosphate precipitation method along with 0.5 µg of pNF-κB-Luc and 8 ng of pRL-TK as described in the figure legends. At 24–36 h after transfection, cells were treated with various concentrations of purified gBs (0–40 µg/ml) for 8 h or left untreated. For positive controls, some samples were stimulated for 8 h with specific TLR ligands (zymosan, 10 µg/ml; LPS, 100 ng/ml; poly(I:C), 10 µg/ml). For negative controls, some samples were stimulated for 8 h with purified HA (20 µg/ml) or purified supernatant of pAcGP67-B. Following stimulation, cells were lysed with 200 µl of Passive Lysis Buffer, and firefly and *Renilla* luciferase activities were assayed using the Dual-Glo luciferase assay system (Promega) according to the manufacturer’s instructions. Data were normalized for transfection efficiency by measuring *Renilla* luciferase activity and luciferase activity values are expressed as the ratio between the firefly and the *Renilla* luciferase. The results are shown as the mean ± SD of three independent experiments.

### gB and Human TLR2 Blocking Experiment and IκBα Degradation Assay

For the blocking Ab assays, TLR2-tranfected HEK293T cells were transiently transfected with pNF-κB-Luc and pRL-TK reporter vectors as described above. At 24–36 h after transfection, cells were blocked with anti-TLR2 mAb or its isotype control Ab IgG2a at a final concentration of 10 µg/ml for 1 h at 37°C prior to stimulation. Subsequently, the cells were treated with gBs (20 µg/ml), heat-denatured (HD) gBs (HD-gBs, 100°C for 15 min) (20 µg/ml), ultraviolet irradiated WT HSV-1 (UV-HSV-1) (MOI = 5) or zymosan (10 µg/ml) for 8 h or left untreated as indicated in the figure legends. When indicated, gBs, HD-gBs and UV-HSV-1 were preincubated with anti-gB mAb or mouse IgG at a final concentration of 10 µg/ml for 1 h at 37°C before addition to cells. After treatment, cell lysates were prepared for the assays. (1) neutralization of gBs-, UV-HSV-1- and TLR2-mediated activation of reporter gene activity was determined using the Dual-Glo luciferase assay system as described above. Data were normalized for transfection efficiency by measuring Renilla luciferase activity and luciferase activity values are expressed as the ratio between the firefly and the Renilla luciferase. The results are shown as the mean ± SD of three independent experiments. (2) Neutralization of gBs-, UV-HSV-1- and TLR2-mediated endogenous IκBα degradation and actin levels were analyzed by IB as described previously [26] using anti-IκBα and anti-actin Abs. Densitometry was performed using the Quantity One 4.4 software system (Bio-Rad Laboratories).

### IL-8 Analysis

To model human monocytes, the THP-1 cell line, which constitutively expresses TLR2 [28], was applied. Cells were seeded into 96-well plates overnight. Then, the cells were incubated with blocking anti-TLR2 mAb or IgG2a isotype control Ab (10 µg/ml) for 1 h at 37°C before treatment with gBs (20 µg/ml), zymosan (10 µg/ml), anti-gB mAb or mouse IgG (10 µg/ml) preincubated gBs (37°C for 1 h) or left untreated as indicated in the figure legends. After 16 to 18 h stimulation, cell-free supernatants from control and treated samples were harvested to determine the secretion level of IL-8 by ELISA analysis using the commercially available BD Human Inflammation Cytometric Bead Array Kit according to the manufacturer’s directions. Data are shown in picograms per milliliter as mean ± SD of two independent experiments.

## Results

### Biological Activity of the Soluble gB

**Figure 1:**
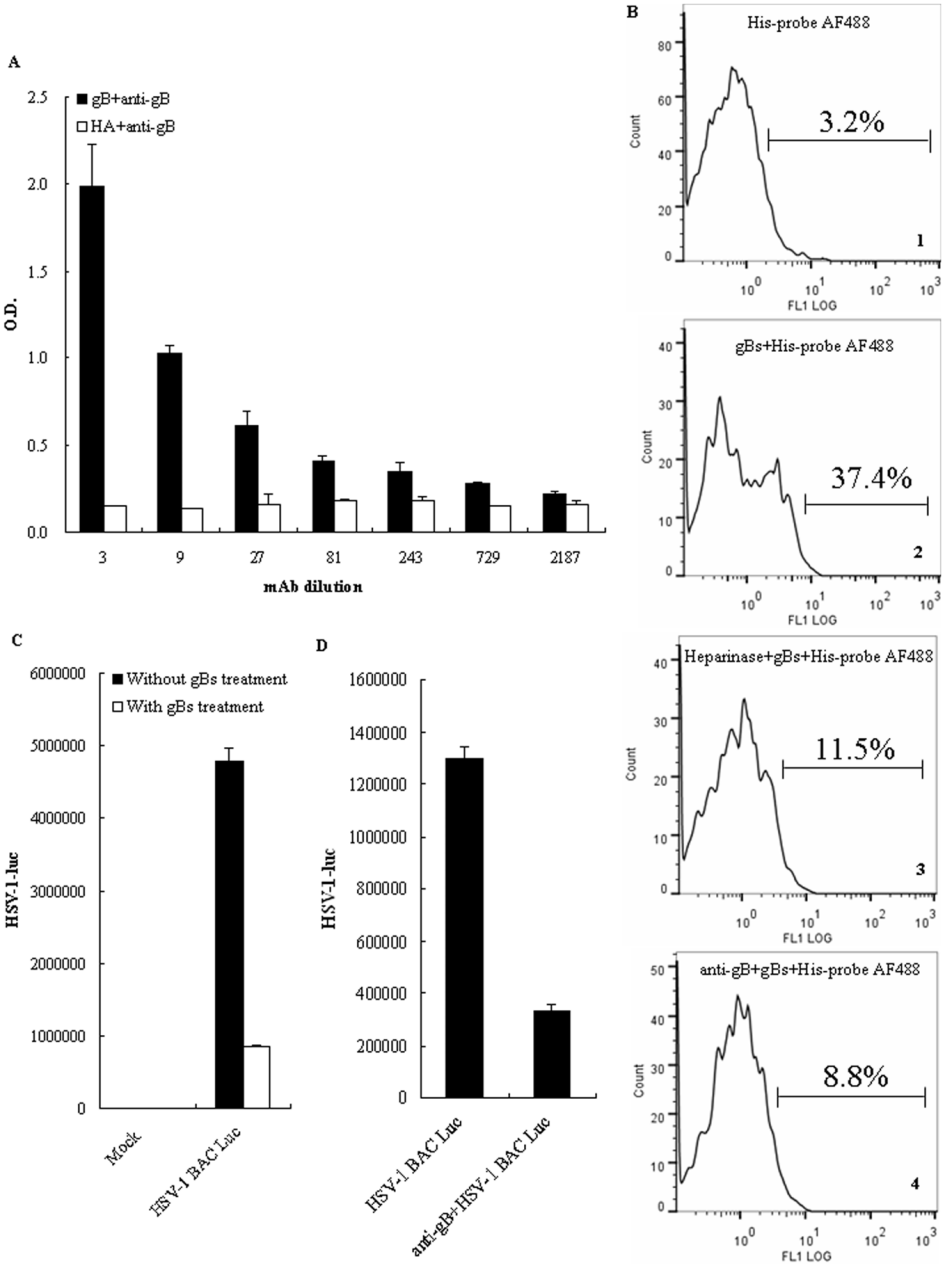
Soluble gB is a biologically active form of the glycoprotein. (A) ELISA reactivity of gBs to anti-gB mAb. gBs or HA, as a negative control, was immobilized on 96-well plates and allowed to react with increasing dilutions of anti-gB mAb. Reactivity was detected by anti-mouse Ab conjugated to peroxidase, followed by tetramethyl benzidine and reading of the optical density (O.D.) at 450 nm. The results are shown as the mean ± SD of two independent experiments. (B) Flow cytometric analysis of the binding of gBs to cells. One experiment representative of two is shown. HEK293T cells were incubated with gBs (20 µg/ml), followed by stained with His-probe AF488 Ab. Panel 1 represents the negative control (cells incubated with His-probe AF488 Ab alone), panel 2 represents the fluorescence of cells incubated with gBs and His-probe AF488 Ab, panel 3 represents the fluorescence of cells treated with heparinase prior to staining with gBs and His-probe AF488 Ab, and panel 4 represents the fluorescence of cells incubated with anti-gB mAb preincubated gBs and His-probe AF488 Ab. Lateral axis, fluorescence intensity; vertical axis, relative number of cells. The values shown above the curves represent the percentages of cells positive for staining. (C) Luciferase activity analysis of inhibition of viral entry by gBs. HEK293T cells were incubated with purified gBs (20 µg/ml) for 2 h or left untreated, followed mock infected or infected with HSV-1 BAC Luc at an MOI of 1 for 24 h. (D) Luciferase activity analysis of inhibition of viral entry by anti-gB mAb. HEK293T cells were infected with HSV-1 BAC Luc or anti-gB mAb (at a final concentration of 10 µg/ml) preincubated HSV-1 BAC Luc (37°C for 1 h) at an MOI of 1 for 24 h, and firefly luciferase activity was measured as described in *Materials and Methods*. The results are shown as the mean ± SD of two independent experiments.

To test whether the recombinant gB protein produced from insect cells is biologically active, we measured its reactivity to anti-gB mAb by ELISA and its ability to bind cells by fluorescence-activated cell sorter (FACS). As shown in Fig. 1A, anti-gB mAb bound to gBs in a dose-dependent manner but did not bind HA. Furthermore, by FACS analysis following incubation with gBs, 37.4% of the HEK293T cells were marked with AF488 labeled His-probe Ab (Fig. 1B, gBs+His-probe AF488) and exhibited a strong reactivity. As negative control, only 3.2% of the HEK293T cells were marked with AF488 labeled His-probe Ab (Fig. 1B, His-probe AF488).

It is reported that gB can bind to the cell surface heparan sulfates [29] and other receptors [30]–[31]. To further demonstrate the specificity of gBs binding and whether this binding can be blocked by anti-gB mAb, HEK293T cells were treated with anti-gB mAb preincubated gBs and His-probe AF488 Ab or treated with heparinase prior to incubation with gBs and His-probe AF488 Ab. As results, heparinase treatment (Fig. 1B, Heparinase+gBs+His-probe AF488), as well as the anti-gB mAb blocking (Fig. 1B, anti-gB+gBs+His-probe AF488), caused a dramatic drop in fluorescence.

Biologically active form of the glycoprotein is able to bind to cell surfaces and inhibit virus entry. To test this possibility, HEK293T cells were treated with purified gBs or left untreated and then infected with HSV-1 BAC Luc or anti-gB mAb preincubated HSV-1 BAC Luc viruses. In untreated cells, HSV-1 BAC Luc virus infection resulted in ∼5000000 units of luciferase activity, and gBs treatment caused a dramatic drop in luciferase activity to ∼1000000 units (Fig. 1C), suggesting that gBs binds to cell surface receptors and inhibits HSV-1 BAC Luc virus entry. Moreover, compared to the negative control (HSV-1 BAC Luc), anti-gB mAb incubation also caused a sharp reduction of luciferase activity from ∼1300000 to ∼400000 (Fig. 1D), indicating that the specific anti-gB mAb can block the HSV-1 BAC Luc infection. Taken together, these results indicate that the recombinant gB protein represents a biologically active form of the glycoprotein.

### The HSV-1-encoded gB Interacts with TLR2

**Figure 2:**
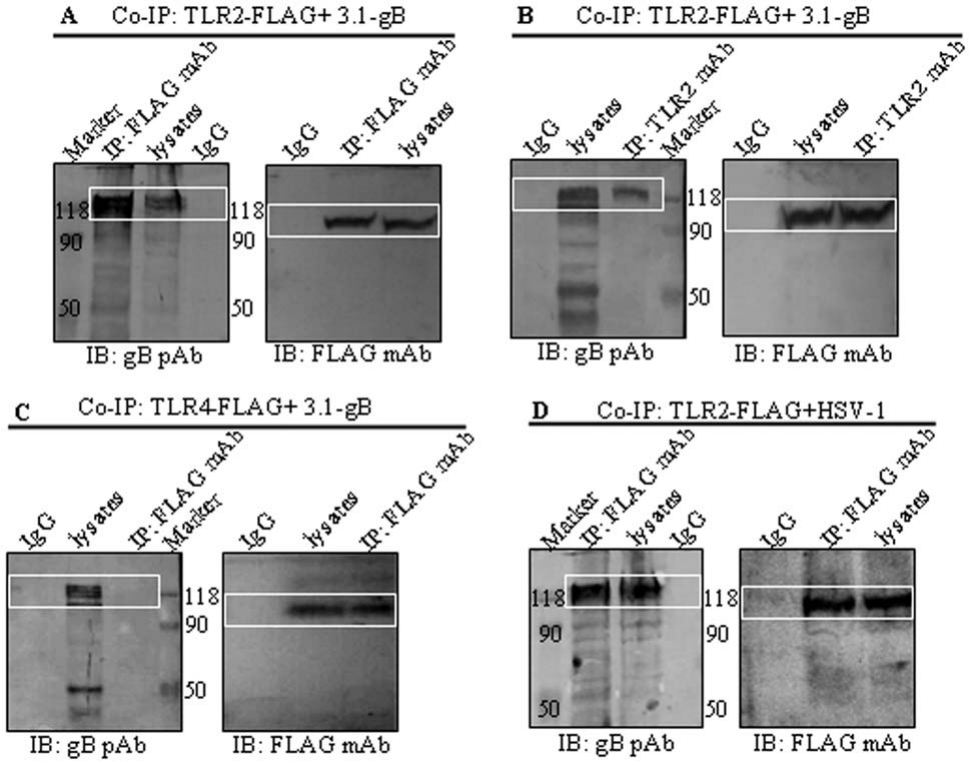
HSV-1-encoded gB coimmunoprecipitates with TLR2 in transfected and infected cells. HEK293T cells were transiently co-transfected with 5 µg of two expressing plasmids pCMV1-FLAG-hTLR2 and pcDNA3-gB (A and B), pCMV1-FLAG-hTLR4 and pcDNA3-gB (C) or first transfected with 5 µg of pCMV1-FLAG-hTLR2 for 24 h followed by infection with HSV-1 at an MOI of 5 (D). Thirty-six hours after transfection or infection, cells were lysed and IP with anti-FLAG mAb, anti-TLR2 mAb or nonspecific mouse antibody (IgG) was performed. Immunoprecipitated proteins, as well as the cell lysates, were separated by denaturing 10% SDS-PAGE and analyzed by IB with anti-FLAG mAb or anti-gB pAb R68 as indicated. IP, immunoprecipitation. IB, immunoblotting.

Envelope glycoproteins that decorate the exterior of the virion are an emerging class of TLR activators [32] and members of the herpesvirus family have been reported to activate NF-kB via TLR2 [13], [15], [33]–[35]. Therefore, we hypothesized that HSV-1-encoded gB might interact with TLR2 to trigger the NF-κB activation. To test this possibility, HEK293T cells were transiently co-transfected with plasmids expressing gB and TLR2-FLAG or TLR4-FLAG and potential protein-protein interaction were determine by co-immunoprecipitation. The results indicated that gB co-precipitated with TLR2 by anti-FLAG antibody (Fig. 2A) or anti-TLR2 antibody (Fig. 2B) but.not co-precipitated with TLR4-FLAG (Fig. 2C). These results demonstrate that gB forms a complex with TLR2 but not TLR4 *in vivo*. To determine whether the gB-TLR2 complex was present in HSV-1-infected cells, HEK293T cells were transiently transfected with plasmid expressing TLR2-FLAG and infected with WT HSV-1, then Co-immunoprecipitate assays were performed with anti-FLAG antibody. As shown in Fig. 2D, gB was also successfully co-immunoprecipitated with TLR2, suggesting that gB may form a complex with TLR2 in HSV-1-infected cells.

### HSV-1-encoded gB Activates TLR2-dependent Signaling

**Figure 3:**
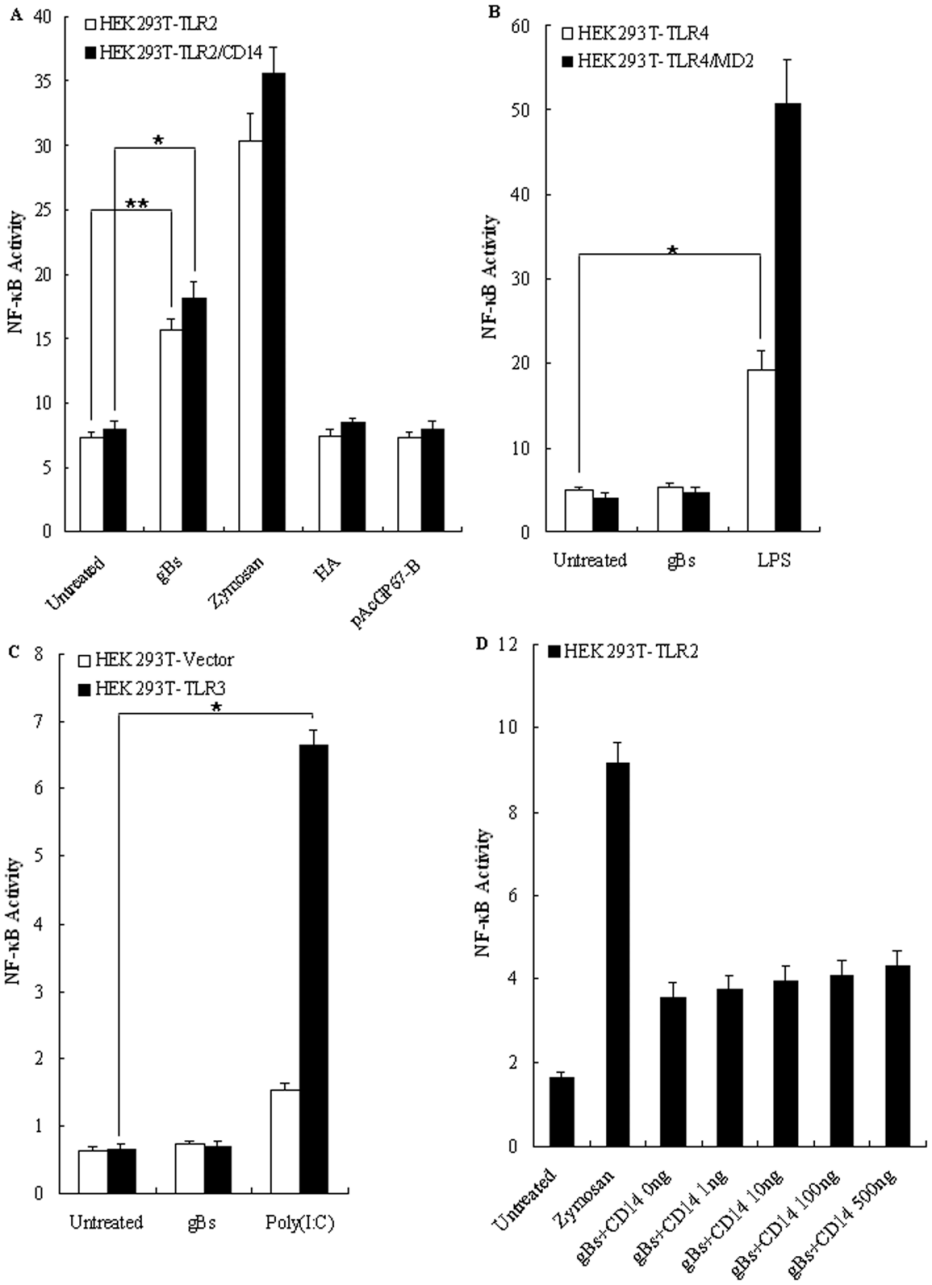
HSV-1-encoded gB stimulates NF-κB activity through TLR2 that does not require CD14. HEK293T cells were transiently transfected with plasmids pCMV1-FLAG-hTLR4, pCMV1-FLAG-hTLR4 plus pEFBOS-HA-hMD2 (B), pTLR3-FLAG, empty vector (C) or pCMV1-FLAG-hTLR2 and NF-κB luciferase reporter plasmid alone or in conjunction with pcDNA3.1-hCD14 (A) or with increasing amounts of plasmid encoding CD14 (D). After 24-36 h, cells were stimulated with zymosan (10 µg/ml), LPS (100 ng/ml), poly(I:C) (10 µg/ml), gBs (20 µg/ml) or purified supernatants from pAcGP67-B and HA protein (20 µg/ml) of influenza A virus or left untreated for 8 h, and luciferase reporter gene activity was measured as described in *Materials and Methods*. Luciferase activity values are expressed as the ratio between firefly and *Renilla* luciferase. The results are shown as the mean ± SD of three independent experiments. Statistical analysis was performed using Student’s *t* test. (A) **, *P = *0.010; *, *P = *0.017. (B) *, *P = *0.045. (C) *, *P = *0.044.

To determine whether the interaction between HSV-1-encoded gB and TLR2 could induce TLR2-dependent NF-κB activation, HEK293T cells, which constitutively express TLR1, TLR6, and TLR10 but do not express TLR2 and TLR4 [36], were transiently cotransfected with vectors encoding human TLR2, TLR4, TLR4/MD2 (a coreceptor of TLR4), TLR3 or empty vector along with an NF-κB luciferase reporter plasmid and the internal control pRL-TK, followed by 8 h of treatment with zymosan, poly(I:C), LPS, purified gBs, HA or supernatant from pAcGP67-B. NF-κB activation, indicated by luciferase reporter activity driven by the NF-κB promoter, was observed in HEK293T-TLR2 cells stimulated with the TLR2 ligand zymosan (Fig. 3A), indicating the functional expression of TLR2. As negative control, recombinant infuenza A virus HA protein purified from insect cells and the supernatant from mock baculovirus infected insect cells (pAcGP67-B) did not trigger the NF-κB activation (Fig. 3A), suggesting that the purified gBs was not contaminated with peptides, lipopolysaccharides or lipopeptides, all of which have been demonstrated to have TLR2 activating properties. Treatment of TLR4-transfected HEK293T (HEK293T-TLR4) cells or TLR4/MD2-transfected HEK293T (HEK293T-TLR4/MD2) cells with gBs did not activate the NF-κB promoter (Fig. 3B). Conversely, treatment of HEK293T-TLR4 and HEK293T-TLR4/MD2 cells with LPS, a ligand for TLR4, resulted in the activation of NF-κB (Fig. 3B), indicating that the purified gB protein does not contain LPS. Likewise, poly(I:C), a ligand for TLR3, but not gBs, triggered NF-κB activation in HEK293T-TLR3 cells (Fig. 3C), indicating no viral dsRNA was present in the gBs.

**Figure 4:**
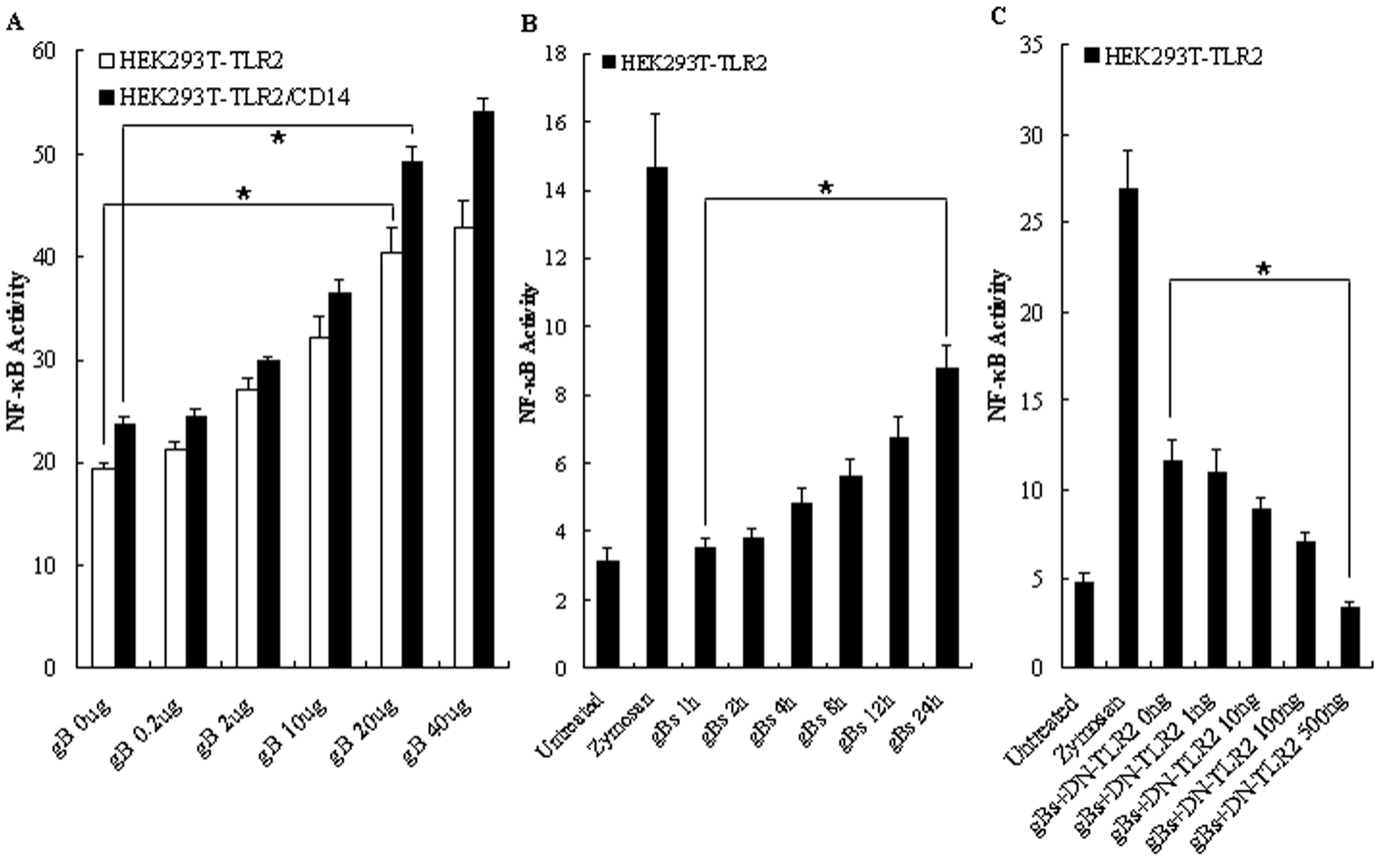
TLR2 is required for gB-mediated NF-κB activation. (A) Dose-dependent activation of NF-κB by HSV-1-encoded gB. HEK293T cells were transiently transfected with pCMV1-FLAG-hTLR2 and/or pcDNA3.1-hCD14 along with NF-κB luciferase reporter plasmids. After 24–36 h, cells were stimulated with various concentrations of gBs (0, 0.2, 2, 10, 20 and 40 µg/ml) for 8 h. (B) Time-dependent activation of NF-κB by HSV-1-encoded gB. HEK293T cells were transiently transfected with pCMV1-FLAG-hTLR2 and NF-κB luciferase reporter plasmids. After 24–36 h, cells were stimulated with gBs (20 µg/ml) for 1, 2, 4, 8, 12, and 24 h or zymosan (10 µg/ml) for 8h or left untreated. (C) Dominant-negative TLR2 dose-dependently inhibits HSV-1-encoded gB-mediated activation of NF-κB. HEK293T cells were transiently transfected with pCMV1-FLAG-hTLR2 and the NF-κB luciferase reporter plasmid along with various concentrations of pCMV1-FLAG-DN-hTLR2. After 24–36 h, cells were stimulated with gBs (20 µg/ml) or zymosan (10 µg/ml) or left untreated for 8 h, and luciferase reporter gene activity was measured as described in *Materials and Methods*. Luciferase activity values are expressed as the ratio between firefly and *Renilla* luciferase. The results are shown as the mean ± SD of three independent experiments. Statistical analysis was performed using Student’s *t* test. (A) HEK293T-TLR2, *, *P = *0.030; HEK293T-TLR2/CD14, *, *P = *0.029. (B) *, *P = *0.024. (C) *, *P = *0.043.

As shown in Fig. 4A, different concentration (0, 0.2, 2, 10, 20 and 40 µg/ml) of gBs treatment of HEK293T-TLR2 leaded to the NF-κB activation in a dose-dependent fashion. Since only 20 and 40 µg/ml but not 2 or 10 µg/ml of gB can activate NF-κB via TLR2, we performed the following experiments with the gB moderate concentration of 20 µg/ml. As shown in Fig. 4B, NF-κB activation by gBs was also found to be time dependent. To further confirm that gB induces the activation of NF-κB via TLR2, dose-response experiments in which HEK293T-TLR2 cells were transfected with various concentrations of vector encoding DN-TLR2 were performed. As shown in Fig. 4C, the activation level of NF-κB by gBs was indeed decreased with increasing concentrations of DN-TLR2 expression plasmid. Taken together, these results suggest that the HSV-1-encoded gB has the capacity to induce the activation of NF-κB in a TLR2-, but not a TLR3- or TLR4-dependent manner, consistent with the previous study that HSV-1 could activate NF-κB through TLR2, but not TLR3 or TLR4 [37].

### Anti-gB and anti-TLR2 Antibodies Blocked TLR2-dependent, gB-mediated Activation of NF-κB

**Figure 5:**
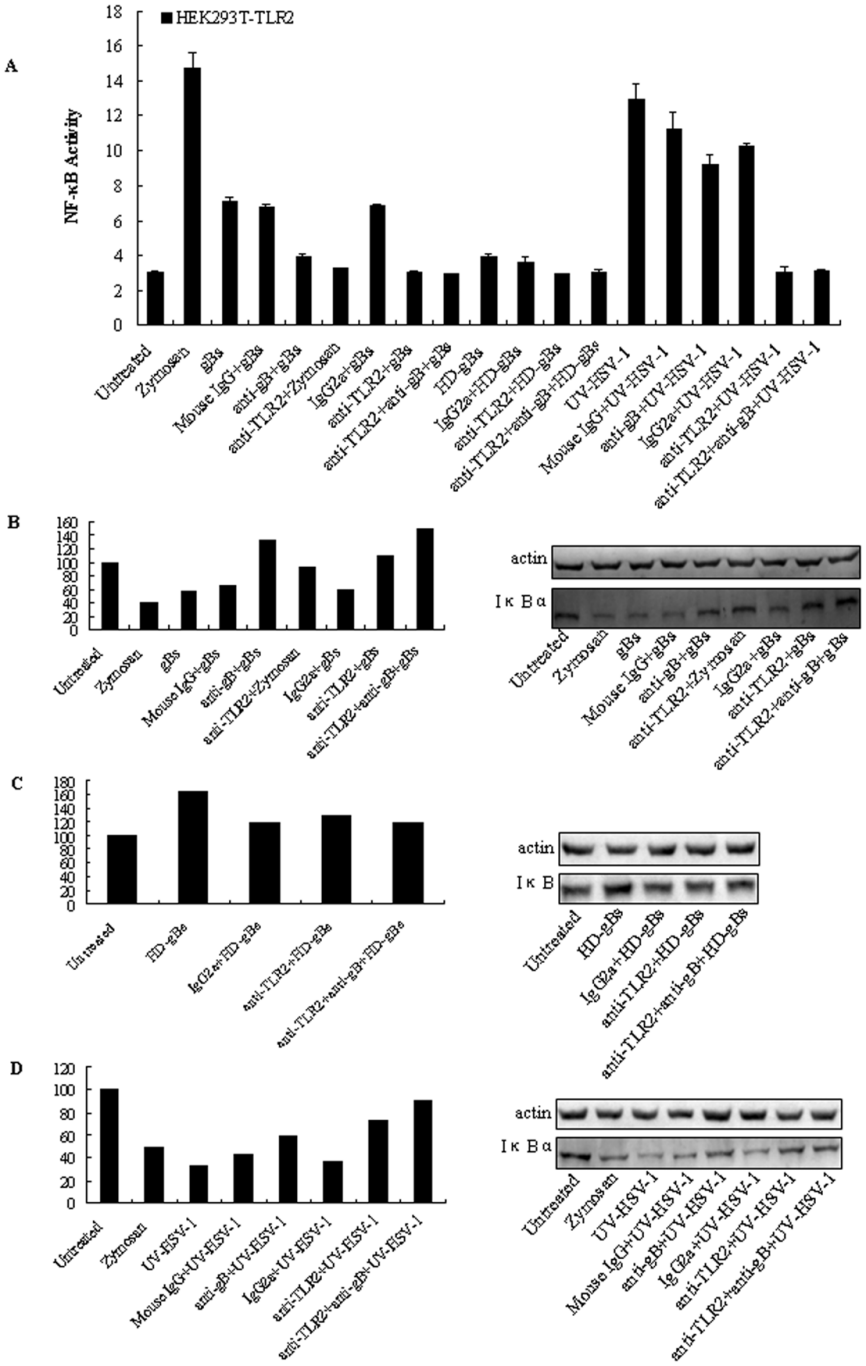
Antibody blocking abrogates NF-κB activation by the HSV-1-encoded gB. (A) HEK293T cells were transiently transfected with pCMV1-FLAG-hTLR2 along with the NF-κB reporter gene plasmid. After 24–36 h, cells were pre-incubated with blocking anti-TLR2 mAb or IgG2a isotype control Ab at a final concentration of 10 µg/ml for 1 h at 37°C followed by treatment with either gBs (20 µg/ml), HD-gBs (20 µg/ml), UV-HSV-1 (MOI = 5), zymosan (10 µg/ml) or anti-gB mAb or mouse IgG preincubated gBs, HD-gBs and UV-HSV-1 at a final concentration of 10 µg/ml (37°C for 1 h) for 8 h, and luciferase reporter gene activity was measured as described in *Materials and Methods*. Luciferase activity values are expressed as the ratio between firefly and *Renilla* luciferase. The results are shown as the mean ± SD of three independent experiments. (B) to (D) Validation of NF-κB activation by gBs and UV-HSV-1 through detection of IκBα degradation. HEK293T cells transfected with TLR2 expressing plasmid for 24–36 h were pre-incubated with blocking anti-TLR2 mAb and IgG2a isotype control Ab at a final concentration of 10 µg/ml for 1 h at 37°C followed by treatment with either gBs (20 µg/ml) (B), HD-gBs (20 µg/ml) (C), UV-HSV-1 (MOI = 5) (D), zymosan (10 µg/ml) or anti-gB mAb or mouse IgG preincubated gBs (B), HD-gBs (C) and UV-HSV-1 (D) at a final concentration of 10 µg/ml (37°C for 1 h) as indicated for 8 h, then cell lysates were prepared and IκBα and actin levels were determined by 10% SDS-PAGE analysis followed by IB using anti-IκBα and anti-actin Abs (right panel). Left panel, The immunoblot shown in the right panel was subjected to densitometric analysis using Quantity One 4.4 software. The intensity of the IκBα bands for each sample was normalized to the intensity of the corresponding actin band. For each lane, quantification is expressed as the ratio of IκBα to actin. IB, immunoblotting. The results are indicative of two independent experiments.

To conclusively demonstrate that the activation of NF-κB is mediated by HSV-1-encoded gB and dependent on TLR2, blocking experiments using anti-gB mAb and anti-TLR2 mAb or mouse IgG and isotype control Ab were performed. HEK293T cells were transiently transfected with TLR2 expression vector, pNF-κB-Luc and pRL-TK plasmids and incubated in the presence of blocking anti-TLR2 mAb or IgG2a isotype control Ab, before treatment of cells with gBs, HD-gBs, UV-HSV-1, zymosan, anti-gB mAb or mouse IgG preincubated gBs, HD-gBs and UV-HSV-1 for an additional 8 h. As shown in Fig. 5A, while IgG2a preincubated cells stimulation with gBs and UV-HSV-1 but not HD-gBs, or cells stimulated with mouse IgG preincubated gBs and UV-HSV-1 led to the activation of NF-κB, pretreatment of HEK293T-TLR2 cells with blocking anti-TLR2 mAb or gBs incubated with anti-gB mAb significantly inhibited HSV-1-encoded gB-mediated activation of NF-κB. Furthermore, blocking anti-gB mAb reduced the TLR2-dependent NF-κB activation meditated by UV-HSV-1, suggesting that the HSV-1-encoded gB is able to activate NF-κB in a TLR2-dependent fashion. However, NF-κB activation is only partly blocked, suggesting that HSV-1 gH and gL [17] or other unknown HSV-1 proteins might also induce the NF-κB activation. Similarly, pretreatment of HEK293T-TLR2 cells with anti-TLR2 mAb resulted in the blockade of NF-κB promoter activity by zymosan. In addition, degradation of IκBα as a marker of NF-κB activation also confirmed the aforementioned results (Fig. 5B, to 5D). Collectively, these data further support the hypothesis that the activation of NF-κB by HSV-1-encoded gB is mediated through TLR2.

### CD14 is not Required for TLR2-dependent gB-mediated Activation of NF-κB

The CD14 protein, a common co-receptor, has been shown to facilitate ligand binding and TLR2 signaling efficacy [15], [38]–[39]. In order to evaluate whether triggering of TLR2 by HSV-1-encoded gB requires the presence of CD14, HEK293T cells, which are known to lack endogenous CD14 expression [40], were transiently cotransfected with plasmids expressing TLR2 and/or CD14 (TLR2/CD14) along with pNF-κB-Luc and pRL-TK plasmids, then stimulated with gBs or zymosan or left unstimulated. As shown in Fig. 3A, NF-κB activation by gBs (20 µg/ml) was clearly measured in HEK293T-TLR2 cells. However, the activation was not significantly enhanced by the presence of CD14 (Fig. 3A and 3D), and the difference between HEK293T-TLR2 cells and HEK293T-TLR2/CD14 cells stimulated with gBs was statistically significant. Since CD14 shifts the dose response to ligand, high concentration of gB might block an effect of CD14, experiments of HEK293T-TLR2/CD14 cells stimulated with distinct amount of gBs were conducted. As shown in Fig. 4A, there is no significant difference of NF-κB activation between HEK293T-TLR2 cells and HEK293T-TLR2/CD14 cells stimulated with gBs at low concentrations (0, 0.2, 2 and 10 µg/ml), suggesting that CD14 is not required for HSV-1-encoded gB-mediated TLR2 activation, which is consistent with the fact that CD14 is not required for HSV-1 recognition by TLR2 [16].

### HSV-1-encoded gB is Predominantly Recognized through TLR2 and TLR6

**Figure 6:**
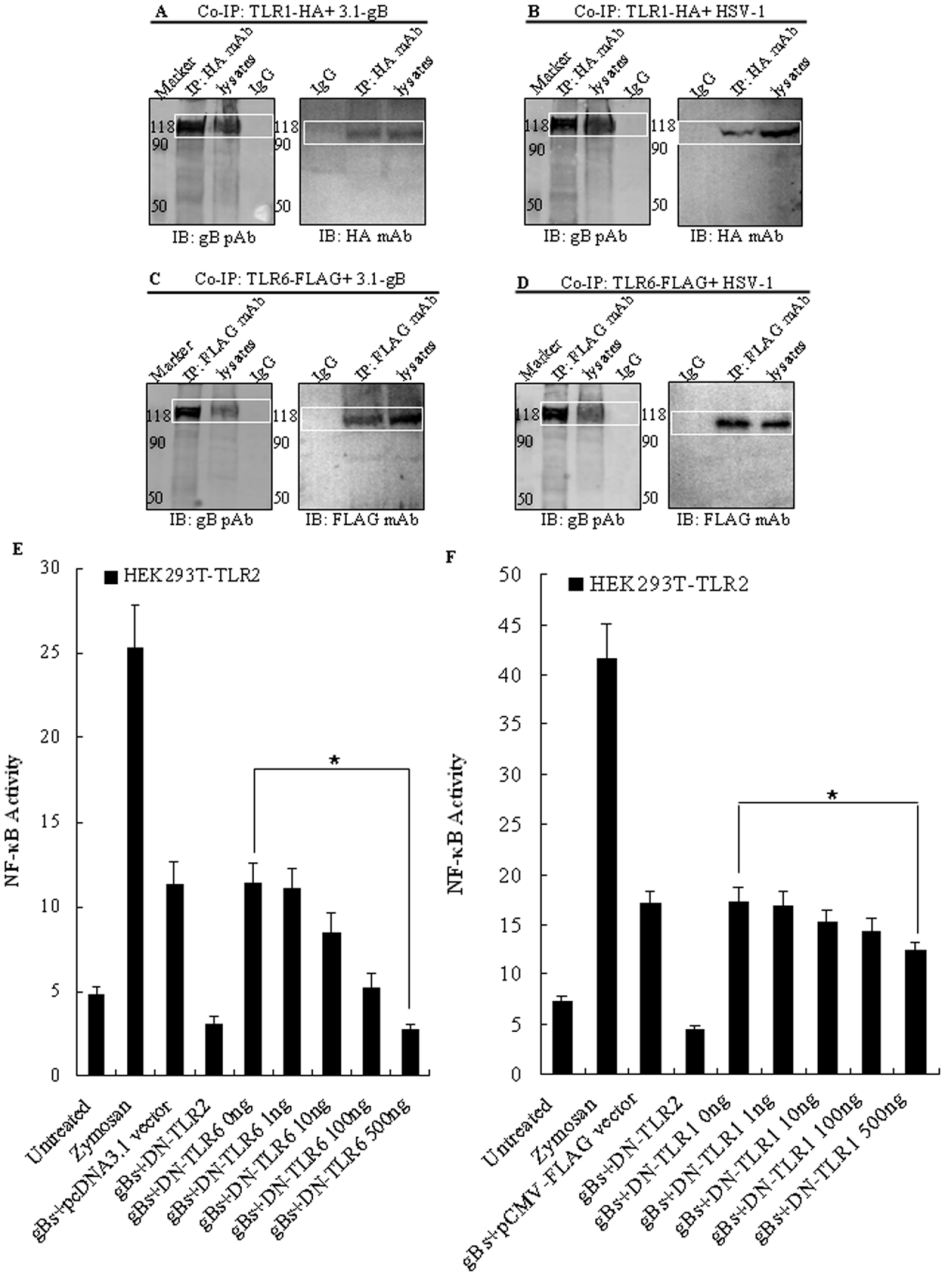
HSV-1-encoded gB activates NF-κB activation through TLR2 in combination with TLR6, but not TLR1. (A) to (D) HSV-1-encoded gB associates with TLR1 and TLR6 independent of TLR2. HEK293T cells were transiently co-transfected with 5 µg of two expressing plasmids pcDNA3-gB and pEFBOS-hTLR1-HA (A), pcDNA3-gB and pFLAG-TLR6 (C) or first transfected with 5 µg of pEFBOS-hTLR1-HA (B) or pFLAG-TLR6 (D) for 24 h followed by infection with HSV-1 at an MOI of 5. Thirty-six hours after transfection or infection, cells were lysed and IP with anti-HA mAb or anti-FLAG mAb or nonspecific mouse antibody (IgG) was performed. Immunoprecipitated proteins, as well as the cell lysates, were separated by denaturing 10% SDS-PAGE and analyzed by IB with anti-HA mAb, anti-FLAG mAb or anti-gB pAb R68 as indicated. IP, immunoprecipitation. IB, immunoblotting. (E) and (F) The activation of NF-κB by HSV-1-encoded gB is mainly mediated by TLR2 and TLR6, and to a lesser extent by TLR2/TLR1 heterodimers. HEK293T cells were transiently transfected with pCMV1-FLAG-hTLR2 and the NF-κB luciferase reporter plasmid along with pCMV1-FLAG-DN-hTLR2, vector (pcDNA3.1 or pCMV-FLAG) and various concentrations of pcDNA3.1-DN-hTLR6 (E) or pCMV1-FLAG-DN-hTLR1 (F). After 24–36 h, cells were stimulated with gBs (20 µg/ml) or zymosan (10 µg/ml) or left untreated for 8 h, and luciferase reporter gene activity was measured as described in *Materials and Methods*. Luciferase activity values are expressed as the ratio between firefly and *Renilla* luciferase. The results are shown as the mean ± SD of three independent experiments. Statistical analysis was performed using Student’s *t* test. (E) *, *P = *0.025. (F) *, *P = *0.031.

*In vivo*, TLR2 functions as a heterodimer with its coreceptors, TLR1 or TLR6 [41]–[42]. Thus far, we have demonstrated the ability of HSV-1-encoded gB to trigger NF-κB activation in HEK293T-TLR2 cells. To determine whether TLR1 or TLR6 interacts with gB. Co-IP experiments were performed in gB-transfected or HSV-1-infected HEK293T cells. As shown in Fig. 6, both TLR1 (Fig. 6A and 6B) and TLR6 (Fig. 6C and 6D) coprecipitated with gB in transfected and infected cells, whereas gB could not be co-immunoprecipitated by mouse IgG in cells expressing gB and TLR1-HA or TLR6-FLAG, suggesting that gB may also form interaction complexs with TLR1 and TLR6 in HSV-1-infected cells in the absence of TLR2.

To further examine the requirement for TLR1 and TLR6 in TLR2-mediated recognition of HSV-1-encoded gB, HEK293T cells were transiently transfected with TLR2, pNF-κB-Luc and pRL-TK plasmids along with DN-TLR2 expressing plasmid, vector (pcDNA3.1 or pCMV-FLAG) and various concentrations of either the DN-TLR1 or DN-TLR6 expression construct. Following transfection, cells were left untreated or treated with gBs or zymosan, and analyzed for luciferase reporter gene activity. As shown in Fig. 6, when compared with DN-TLR2, the expression of DN-TLR6 (Fig. 6E), and to a lesser degree, DN-TLR1 (Fig. 6F), resulted in the reduction of NF-κB activation in HEK293T-TLR2 cells triggered with gBs. These data suggest that the recognition of HSV-1-encoded gB may mostly mediated by TLR2 and TLR6 heterodimer, and to a lesser extent by TLR2 and TLR1 heterodimer.

### The HSV-1-encoded gB Induced NF-κB Activation is MyD88/TRAF6-dependent

**Figure 7:**
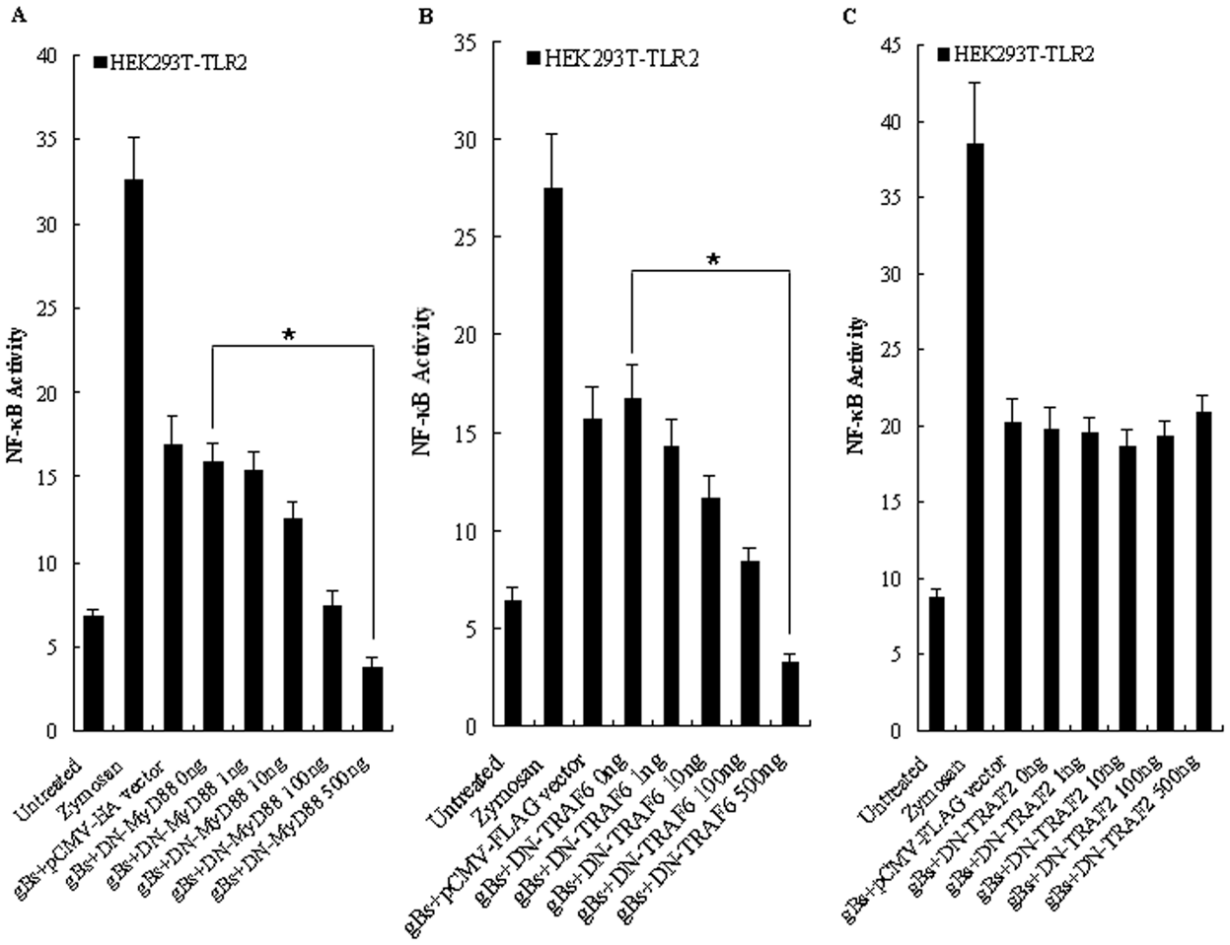
HSV-1-encoded gB signals through MyD88/TRAF6, but not TRAF2. HEK293T cells were transiently transfected with pCMV1-FLAG-hTLR2 and the NF-κB luciferase reporter plasmid along with vector (pCMV-HA or pCMV-FLAG) and various concentrations of pHA-DN-MyD88 (A), pDN-TRAF6 (B) or pFLAG-DN-TRAF2 (C). After 24–36 h, cells were stimulated with gBs (20 µg/ml) or zymosan (10 µg/ml) or left untreated for 8 h, and luciferase reporter gene activity was measured as described in *Materials and Methods*. Luciferase activity values are expressed as the ratio between firefly and *Renilla* luciferase. The results are shown as the mean ± SD of three independent experiments. Statistical analysis was performed using Student’s *t* test. (A) *, *P = *0.011. (B) *, *P = *0.023.

MyD88 is a crucial adaptor protein that is downstream of TLR activation, whereas TRAF6 (tumor necrosis factor receptor-associated factor 6) is a signal transducer in the NF-κB pathway, both of which are essential for the production of inflammatory cytokines [33], [34], [43]. We next examined whether the activation of NF-κB by HSV-1-encoded gB involves the adaptor molecules MyD88 and TRAF6. When HEK293T-TLR2 cells were cotransfected with various concentrations of DN-MyD88 or DN-TRAF6 expression plasmid followed by 8 h of treatment with gBs, we found that overexpression of the DN-MyD88 (Fig. 7A) or DN-TRAF6 (Fig. 7B) prevented the activation of NF-κB by gBs in a dose-dependent fashion. As negative control, overexpression of the vector (pCMV-HA or pCMV-FLAG) or the DN form of the intracellular mediator TNF receptor-associated factor 2 (TRAF2), which is not involved in the TLR signaling pathways, did not result in the reduction of NF-κB activation (Fig. 7C). Altogether, these data suggest that the HSV-1-encoded gB-mediated activation of NF-κB is TLR2/MyD88/TRAF6 signal mediated, consistent with the work by Mansur *et al.* shown that the MyD88-dependent signaling pathway is essential for HSV-1 induced chemokine and cytokine responses [35].

### HSV-1-encoded gB Induces Inflammatory Cytokine Production in Human THP-1 Monocytes Dependent on TLR2

**Figure 8:**
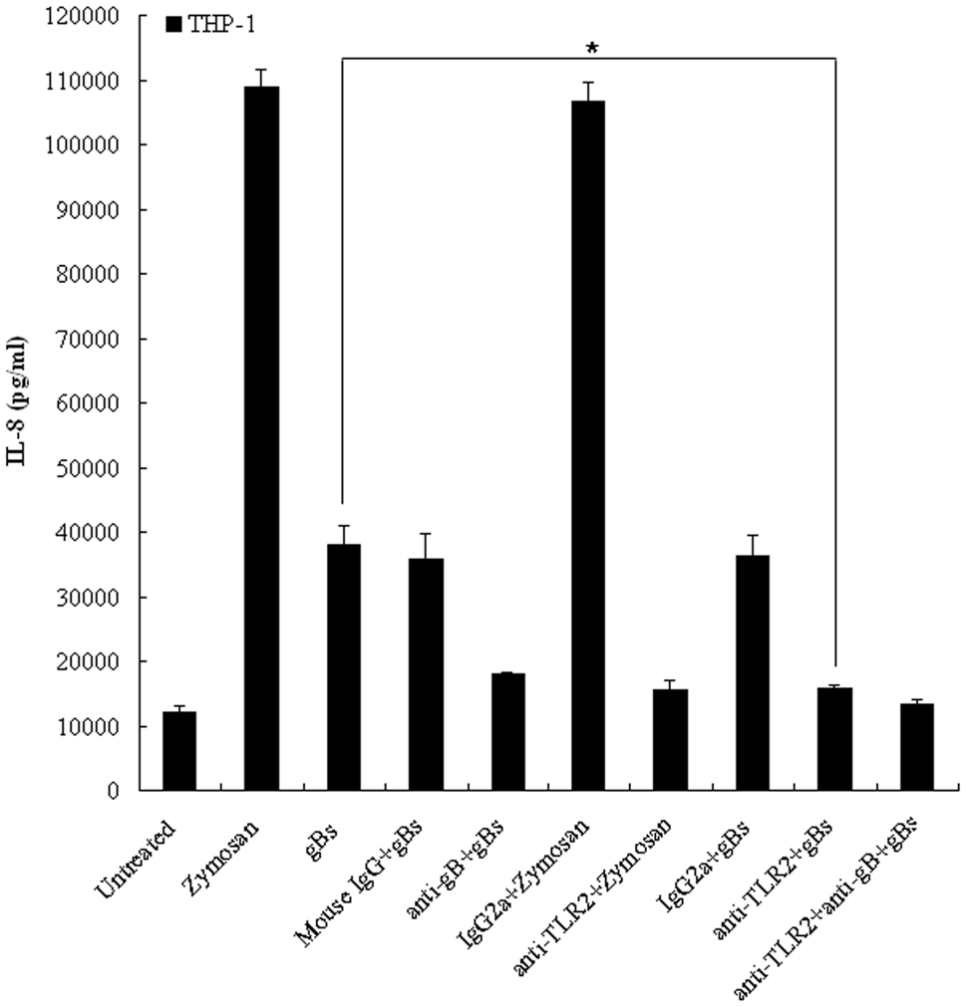
HSV-1-encoded gB-mediated cytokine secretion in the human monocytic cell line THP-1 is TLR2 dependent. THP-1 cells were incubated with blocking anti-TLR2 mAb or its IgG2a isotype control Ab at a final concentration of 10 µg/ml for 1 h at 37°C followed by treatment with either gBs (20 µg/ml), zymosan (10 µg/ml) or anti-gB mAb or mouse IgG preincubated gBs at a final concentration of 10 µg/ml (37°C for 1 h). After 16–18 h, cell culture supernatants from control and treated samples were collected and analyzed for IL-8 production using the BD Human Inflammation Cytometric Bead Array Kit as described in *Materials and Methods*. The results are shown as the mean ± SD (picograms per milliliter) of two independent experiments. Statistical analysis was performed using Student’s *t* test. *, *P = *0.035.

To establish whether the HSV-1-encoded gB has the competence to elicit inflammatory cytokine responses through TLR2 signaling in human monocytes, antibody blocking experiments were performed to block interactions between gB and TLR2 on the surface of THP-1 cells. THP-1 cells were incubated with anti-TLR2 mAb for 1 h followed by treatment with gBs, zymosan, anti-gB mAb or mouse IgG preincubated gBs. After 16–18 h, cell culture supernatants from control and stimulated samples were collected and the release of IL-8, which was used as a representative marker of inflammatory cytokine activation, was measured by ELISA. The TLR2-specific control, zymosan, induced IL-8 secretion from THP-1 cells, which was inhibited by the addition of anti-TLR2 blocking mAb but not control IgG2a. Treatment with gBs induced IL-8 production in THP-1 cells and was dramatically attenuated with the anti-gB and anti-TLR2 blocking Abs (Fig. 8). Taken together, these data further suggest that HSV-1-encoded gB is interacting with cell surface receptor TLR2 to elicit inflammatory cytokine secretion.

## Discussion

During the past few years, a number of structural components of different viruses have been reported to activate TLR2 and induce inflammatory cytokine production [8]–[11]. Furthermore, different members of the human herpesvirus family were also found to activate inflammatory responses via TLR2 [15], [37], [39]. Several studies have shown that TLR2 is activated by an HSV-1 component(s) [16], [37], [44]. Notably, a common characteristic of all of the herpesvirus envelope glycoproteins is that they have important roles in mediating binding and/or penetration into susceptible cells [45]–[46]. Each viral envelope glycoprotein, which is able to interplay with one or more cellular receptors, is a compelling target for the TLR system, as they are the first component of the virus to make contact with the cell. Consequently, rapid detection of viral envelope glycoproteins would allow the host to set the innate defense in motion at the earliest stages of infection, perhaps even before the virus enters the cell. The viral envelope glycoprotein gB is highly conserved throughout the herpesvirus family [47], and it is well established that HSV-1-encoded envelope glycoprotein gB is essential for virus-cell fusion events [48]. Therefore, we speculated that the HSV-1-encoded gB may also be recognized by the TLR system.

HSV-1 is known to induce the activation of NF-κB. In this study, we demonstrate that the HSV-1 envelope glycoprotein, gB, induces NF-κB activation in transfected HEK293T cells in a time- and dose-dependent fashion through TLR2, and this activation requires the recruitment of the adaptor molecules MyD88 and TRAF6, whereas Leoni and colleagues have shown that the HSV-1-encoded gB is the TLR2 ligand but could not activate the TLR2 signaling [17], these different results might due to various protein expressing systems and purification styles. It is reported that TLR2 may form a heterodimer with its co-receptor TLR1 or TLR6 for signaling [5]. Our aforementioned Co-IP assays suggest the interaction between HSV-1-encoded gB and TLR2. gB also coimmunoprecipitates with TLR1 and TLR6, suggesting the interactions between these receptors and gB. However, these interactions could not lead to the activation of NF-κB, since the activity of NF-κB promoter is not activated in HSV-1-encoded gB-stimulated WT HEK293T cells (Fig. 3C and data not shown), indicating HSV-1-encoded gB can interact with TLR1 and TLR6 in the absence of TLR2, and these interactions are not sufficient to induce the NF-κB activation. Furthermore, there was no significant effect in the expression levels of the NF-κB reporter gene in HEK293T-TLR2 cells transfected with DN-TLR1, whereas overexpression of DN-TLR6 in HEK293T-TLR2 cells resulted in a decreased activation of the NF-κB reporter gene in a dose-dependent manner following treatment with the HSV-1-encoded gB. These data indicate that the HSV-1-encoded gB may associate primarily with TLR2 and/or TLR6 to mediate TLR2 signaling pathway.

CD14 is considered as a pivotal accessory molecule of the TLR2 complex which participates in TLR2 signaling for eliciting inflammatory cytokine induction [38], [49]. VZV and HCMV are two examples of herpesviruses that activate the production of inflammatory cytokines via TLR2 and CD14 [15], [39]. In an attempt to evaluate whether CD14 is implicated in TLR2 activation by HSV-1-encoded gB, we observed that human CD14 may not participate in the recognition of HSV-1-encoded gB by TLR2, as there was no significant difference in the activation levels of the NF-κB reporter gene by HSV-1-encoded gB in HEK293T-TLR2 cells when compared with that of HEK293T-TLR2/CD14 cells, suggesting that CD14 is not essential for induction of NF-κB and that the composition of the TLR2 complex recognizing HSV-1 particles is different from the complex involved in VZV and HCMV recognition.

It is well established that HSV-1 can productively induce a robust expression and secretion of a wide range of cytokines and chemokines in HSV-1-infected animals and cells [16], [37], [50]. However, except for the gD [51], gH and gL [17] of HSV-1, the viral protein(s) responsible for eliciting the increased production of cytokines and chemokines are not completely understood. Using antibody blocking experiments, we demonstrate conclusively that TLR2 can mediate the NF-κB activation and production of the proinflammatory cytokine IL-8 in response to HSV-1-encoded gB in the monocytic THP-1 cell line. This recognition pathway may play an important role *in vivo* in the pathogenesis of HSV-1 infection, since glycoproteins are highly expressed on infected cells within HSV-1 lesions [20].

TLR activation is a double-edged sword and may either lessen or worsen disease, depending on the pathogen and the location of the infection. Kurt-Jones *et al.* show that in the case of HSV-1, the induction of TLR2-mediated cytokine response in the mice brain contributes to the death of mice [37], suggesting that TLR2 is a pivotal molecule implicated in producing neuroimmune responses through the generation of proinflammatory mediators, which may attribute to the interaction between TLR2 and gB. Therefore, gB could be a potential target for the development of novel therapeutic agents against the neonate encephalitis caused by HSV-1, since many of the pathological processes associated with HSV-1 disease are facilitated or directly mediated by inflammatory cytokines.

Together, the results presented in this study add to the growing body of evidence suggesting that HSV-1 can activate innate immunity during binding and entry into host cells. We propose that envelope glycoprotein gB, already well appreciated for its role as a mediator of virus entry, also interacts with TLR2 during entry to initiate a cellular activation pathway that results in the activation of NF-κB and induction of inflammatory cytokines, which may contribute to the pathology observed in infections caused by HSV-1. Accordingly, inhibition of gB-TLR2 interactions might prove to be an effective approach in the treatment of overzealous neuroimmune responses seen during HSV-induced encephalitis.
